# Effective Doses of Ionizing Radiation during Therapeutic Peat Mud Treatment from a Deposit in the Knyszyn Forest (Northeastern Poland)

**DOI:** 10.3390/ijerph17186819

**Published:** 2020-09-18

**Authors:** Jacek Kapala, Maria Karpinska, Stanislaw Mnich

**Affiliations:** Department of Biophysics, Medical University of Bialystok, Mickiewicza 2A, 15-222 Bialystok, Poland; maria.karpinska@umb.edu.pl (M.K.); mnich@umb.edu.pl (S.M.)

**Keywords:** natural radioactivity, effective radiation dose, peat mud

## Abstract

Radioactivity measurements of 61 therapeutic peat mud samples from the Podsokoldy deposits, near Suprasl, were performed using gamma spectrometry. The authors identified the presence of 13 isotopes with the arithmetic mean of activity (in Bq kg^−1^): ^137^Cs-7, ^40^K-24, ^208^Tl-1, ^212^Bi-3, ^212^Pb-2, ^228^Ac-2, ^210^Pb-33, ^214^Bi-11, ^214^Pb-11, ^226^Ra-53, ^234^Th–47. The effective dose obtained during treatment with 15 peat mud baths (lasting 30 min) was 0.078 μSv. Use of peat mud compresses in the same number and period of exposure to the entire body surface caused absorption of a dose of 0.153 μSv. The authors discuss the probability of tissue radiation from isotopes present in the peat mud. In light of radiobiological knowledge, the therapeutic effect of ionizing radiation during peat mud therapy appears to be very unlikely.

## 1. Introduction

For years, many diseases have been treated with peat mud in balneology. Numerous papers contain observations indicating the ion-exchange characteristics of the organic materials contained in peat mud [1,2,3,4]. Such properties make it possible to bind and exchange significant amounts of ions present in the environment. It seems quite likely that radioactive elements present in the soil environment in the form of ions may bind to a greater extent with organic peat mud substances than the concentrations present in the soil. 

Humic substances constitute a collection of high-molecular-weight polyelectrolytes formed in synthesis reactions from simple elements that arose from previously decomposed organic matter [1,2,3,4,5,6]. Humic acid, fulvic acid, and humins present in peat mud are composed of aromatic cores, to which the following are attached (or sometimes trapped between them): aliphatic chains, functional sugar groups, peptides, and a significant number of carboxylic groups [6,7,8,9,10].

Already in 1904, Prof. Borgman observed the existence of peat mud deposits with increased radioactivity in Russia [11]. Thus, it seems likely that there may be peat deposits with increased binding of positive ions, and hence also radioactive isotopes, which are metals. 

Humic substances form complexes during contact with metals and organic substances. They participate in ion exchange, undergo sorption on mineral surfaces, and participate in redox reactions. Dissociated, negatively charged groups can form ionic bonds with polyvalent metals resulting in the formation of different-sized complexes. Two parameters that mainly affect the ability to bind metals with humic substances are pH and ionic strength. Reports regarding studies on the binding sites of metals with humic acids indicate that the most important groups are the COOH and -OH groups associated with phenols [12,13,14]. Both of these factors affect the spatial conformation of molecules and metal-binding occurring in the environment, even in trace amounts. Despite numerous studies concerning the peat peloids’ mechanism of therapeutic action, current knowledge is fragmentary and limited. The extensive literature review conducted by Kulikowa, from the perspective of proposed mechanisms of action of humic substances, revealed the existence of many contradictory opinions [15]. However, nearly all authors link the pharmacological effects to humic acids [16,17,18]. 

The aim of the study was to determine the natural and artificial radionuclide content in raw peat mud obtained for therapeutic purposes from the Podsokoldy deposits, and to assess patient exposure to ionizing radiation from peat mud treatment.

## 2. Materials and Methods

### 2.1. Deposit Location and Characteristics

**Figure 1:**
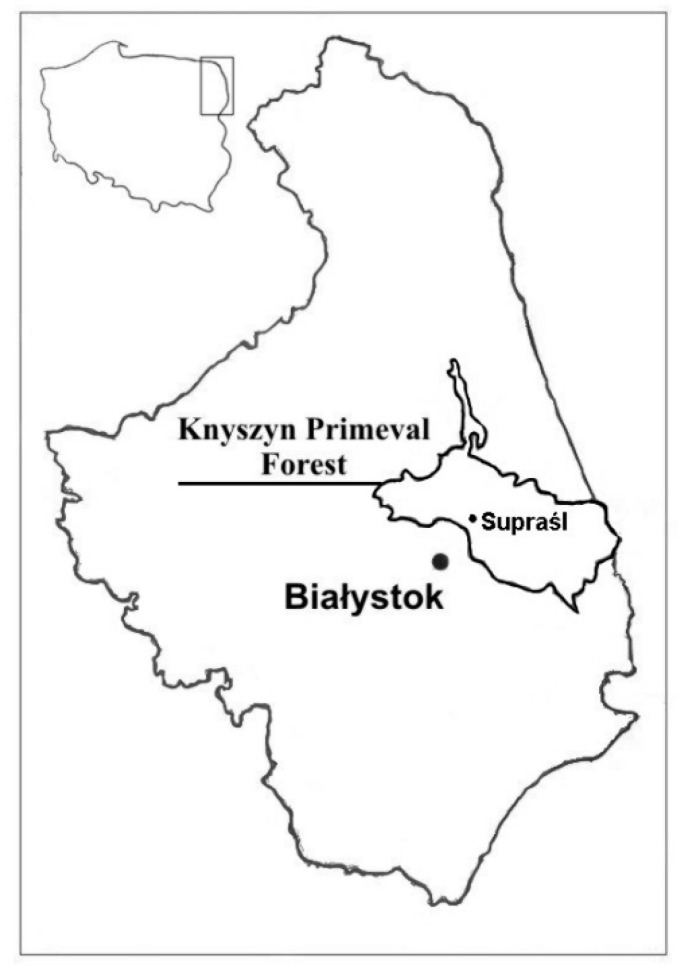
Location of Suprasl.

Suprasl is a lowland climatic and therapeutic peat mud spa town. It lies in the Knyszyn Forest Landscape Park buffer zone, has excellent climatic conditions, and rich, ecologically clean vegetation. The Podsokoldy peat deposits are located about 8.5 km from Suprasl. Geographical coordinates of the middle of the deposit are 53°14′3,8″ latitude, 23°27′8,6″ longitude (Figure 1).

### 2.2. Samples

We examined 61 samples of therapeutic peat mud collected from the Podsokoldy deposits. The geological profile of the deposit was the following: peak formed from peat from a thickness of 0.1 m, below a layer of low peat with a thickness of 2 to 2.7 m, underlain by sand [19]. The Podsokoldy deposits are low-lying peats with a decomposition range from 33.33% to 43.33%. 

The peat samples were collected at seven points within the deposit. At each of the seven locations, cores were collected and divided into 10 cm sections. This divided material of each core was measured by numbering samples from 1 (top layer), giving subsequent numbers with increasing depth. We were not able to collect 10 layers at all the sites, because in a few places, the peat mud deposits were not deep enough. 

### 2.3. Measurement Method

The samples were dried at 105 °C to constant weight, sifted through a sieve of <2 mm so as to remove vegetable material, and placed in Marinelli beakers with a volume of 450 mL. Sample activity was measured using a semiconductor detector by gamma spectrometry. The analysis of the recorded pulses was done with a spectrometric set from CANBERRA with a 34.8% coaxial germanium detector and Genie 2000 computer system for spectral collection and analysis. The applied measurement method used ensures the lower limits of detection (LLD) using Marinelli geometry (450 mL), calculated using the method by Currie (Table 1) [20]. The same measurement time of 160,000 s was used for all samples.

### 2.4. Method for Estimating Dose

It was assumed that peat mud bath fluid consisted half of mud and half of the water that diluted it. This information was obtained from employees servicing bathing stations. It was assumed that during the bath, the patient had 94% of the total body surface area immersed. Mud treatments based on applying the therapeutic peat mud paste onto the skin were also used. Such a preparation usually consists of 80% of peat mud supplemented with water and some peat or herbal extracts. The equivalent and effective dose obtained during such a series of treatments is shown in Table 2. To determine the equivalent dose H from beta radiation, the following equation was used:

H = A T *β*, (1)

where

A—Surface activity in Bq cm^−2^,
T—exposure time in hours,
*β*—conversion ratio Sv h^−1^ to Bq cm^−2^ [21].

The effective dose E from the beta emitters was determined from the dependence

E = H w s,(2)

where

H—equivalent dose,
w—skin weighting factor 0.01,
s—fraction of the surface of immersed skin.

The maximum activity of the specific isotopes determined for peat mud was used for the dose calculation. Thus, the doses are somewhat inflated, but this approach is justified from the point of view of radiological protection. 

This article complied with ethical standards. Ethics approval and consent to participate No human samples were used in this study and all experiments were chemically and in laboratory scale. 

## 3. Results

The specific isotope activity was measured in 61 samples of therapeutic peat mud collected from the Podsokoldy deposits. The registered isotopes beyond ^137^Cs and ^40^K belong to two radioactive series. The four radionuclides belong to the thorium series (^208^Tl, ^212^Bi, ^212^Pb, ^228^Ac), and five to the uranium series (^210^Pb, ^214^Bi, ^214^Pb, ^226^Ra, ^234^Th). 

The statistical parameters of the specific activity of the samples were determined. The obtained values are presented in Table 1. 

**Figure 2:**
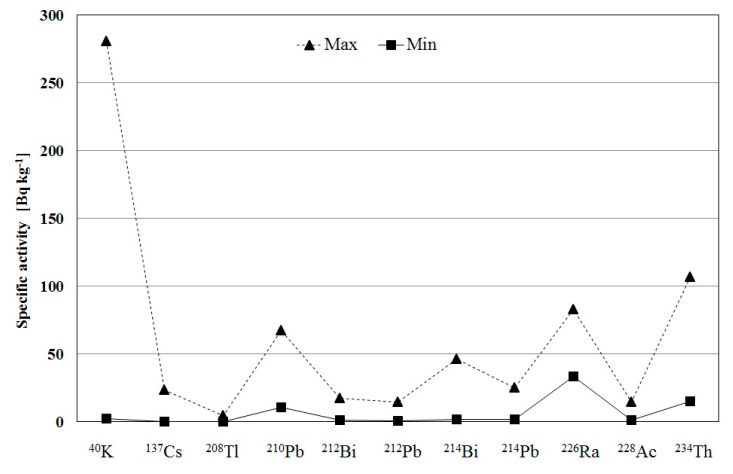
Extreme values of specific activity of radionuclides measured in the collected data.

We determined the total values of specific activity in particular cores, that is, the sum AM of specific activity of individual isotopes located in the entire core. The total specific activities of all isotopes contained in the cores collected from the seven locations ranged from 228 Bq kg^−1^ to 278 Bq kg^−1^. Figure 2 presents the extreme values of the specific activity for all samples.

### Skin Dose and Effective Dose Calculation

The doses obtained during peat mud baths (Table 2) and skin application with mud paste (Table 3) were estimated. 

During the series of bath treatments, a patient received an effective dose from beta emitters of approximately 17.2 nSv, and 61.1 nSv from gamma emitters. In total (from β and γ radiation), through the whole series of bath treatments, a patient received an effective dose of approximately 78 nSv (0.078 μSv). 

The effective dose from the beta emitters contained in the peat mud paste applied to patients during a series of treatments was 33.3 nSv, and 119.8 nSv from gamma emitters. The total dose from emitters (from β and γ radiation) present in the mud paste applied to patients during 15 days of therapy was 153.1 nSv (0.153 μSv). In the case of compress application on selected parts of the body, the dose is accordingly smaller in proportion to the fraction of the surface of the skin covered with the paste in relation to the entire body surface.

## 4. Discussion

Spa treatment of degenerative diseases of the musculoskeletal system is usually a continuation of hospital treatment. One of the treatments used in these diseases is exposure to natural ionizing radiation. The applied treatments are baths and inhalations containing elevated concentrations of radon or drinking mineral waters containing significant amounts of α-emitters [22]. Patients suffering from rheumatoid arthritis treated in the spa town of Bad Brambach (Saxony) with radon baths (1.3 kBq/liter) felt a significant improvement. The authors suspect that the accompanying inhalations of radon reduced the perception of pain [23].

There are few publications describing the effective doses received by patients during balneological procedures as a result of the action of ionizing radiation and its effect on the body. They mainly describe the dosages and effects caused by the radon ^222^Rn [24,25,26,27] or drinking mineral waters [26,28]. In the health resort of Heviz (Hungary), patients received a dose of 81 μSv with ^222^Rn and its derivatives during a two-week treatment with baths and inhalations [26]. Patients with lung diseases during inhalation in the Topolka cave (Hungary) received doses of radon ranging from 0.18 to 4.22 mSv, depending on the duration of therapy [25]. The results obtained are over three times higher than obtained average doses of measured samples from eight Polish spa towns [29].

In the above-described situations, when the specific activity of the isotope entering the body through the alimentary or inhalation route was known, the dosage calculation became clear. All that needed to be done was to apply the conversion factor per the IAEA [30]. ICRP Publication 72 connects total radioactivity to the method of nuclide introduction into the body [31]. In addition, in the case of the inhalation route, exposure time should be considered.

The situation ceases to be so unambiguous in the case of external exposure, when we are dealing with exposure to low-penetrating alpha or beta radiation. It is obvious that radiation only has biological effects if it reaches radiosensitive cells.

According to ICRP, such cells are the basal cells of the epidermis at a depth of 50–100 µm. For dose estimation, ICRP recommends a depth of 70 µm [32,33,34]. Three α-emitters occurred in the measured peat mud samples. All isotopes present in peat mud that emit α-radiation do not have enough energy to reach the radio-sensitive cells. In this case, the alpha radiation in the dose calculation should be omitted.

The effective dose determined from beta and gamma emitters in a series of peat mud baths from the Podsokoldy deposits, amounting to approximately 0.078 μSv, was very small. The doses obtained during mud baths were four orders of magnitude lower than the inhalation doses of radon received at home by the inhabitants of north-eastern Poland (1.8 mSv) [35]. Compared to the routine AP chest X-ray (0.3 mGy) [36], the received dose was over three orders of magnitude smaller. 

Assessment of the biological effects of low doses of ionizing radiation is the subject of research and controversy. The linear dose-effect relationship is accepted by international radiation protection bodies. However, publications describing the beneficial effects of small doses of ionizing radiation on health have appeared for many years. This effect is called radiation hormesis hypothesis in the literature [37,38,39,40,41,42,43]. Most hormesis studies where tissue effects were noted used doses of 40 to 200 mGy [44,45,46,47,48,49,50]. Increased macrophage activity and an increased CD8+ T cell response were observed [44]. Other observations include a decrease in IL-10 cytokine production and stimulation of IL-12 cytokine expression [46], apoptosis reduction [43], and suppression of lung cancer [47].

Studies in the literature present fragmentary explanations of the observed effects, while comprehensive understanding of this phenomenon is quite distant [37,38,39,40,42].

As stated above, the calculated doses a person received during peat mud treatment from the Podsokoldy deposits, which were 0.078 μSv and 0.153 μSv in a series of baths and through skin application, respectively, were about four orders of magnitude less than that given by Tempera (from 0.12 mSv to 0.80 mSv) [27] and about three orders from calculations made by Somlai (81 μSv) [26]. Even assuming the radiation hormesis hypothesis to be true, it is unlikely that such small doses would be able to trigger a hypothetical hormesis mechanism. 

## 5. Conclusions

During treatment based on 15 peat mud baths, patients received an effective dose from beta and gamma emitters of about 0.078 μSv. In the case of a series of treatments consisting of applying peat mud paste onto the skin, the effective dose was approximately 0.153 μSv.

The therapeutic effect of spa treatment with peat mud is most likely caused by other effects than those which can be caused by peat radioactivity.

## Figures and Tables

**Table 1:** The median (M), arithmetic mean (AM), geometric mean (GM), standard deviation (SD), and low limit of detection (LLD) of the specific radioisotope activity measured in all 61 samples.

Radionuclide	M	AM	GM	SD	LLD
	Bq kg^−1^				
^40^K	14	24	15	47	0.6–1.1
^137^Cs	4	7	4	6	0.02–0.11
^208^Tl	1	1	1	1	0.03–0.1
^210^Pb	28	33	28	17	0.46–1.1
^212^Bi	3	3	3	3	0.07–0.11
^212^Pb	2	2	2	2	0.05–0.12
^214^Bi	10	11	10	7	0.06–0.15
^214^Pb	10	11	10	5	0.07–0.3
^226^Ra	50	53	52	13	0,38–1.56
^228^Ac	2	2	2	2	0.09–0.21
^234^Th	40	47	41	25	0.61–1.99

**Table 2:** The equivalent H dose and the effective E dose obtained during a series of 15 mud bath treatment applications to the skin by beta and gamma radiation during half-hour exposures.

Radionuclide	H	E (β)	E (γ)
	[nSv]		
^40^K	891.5	8.5	41.4
^137^Cs	40.2	0.4	1.3
^208^Tl	87.9	0.8	1.7
^210^Pb	0	0	0.2
^212^Bi	80.7	0.8	1.4
^212^Pb	16.5	0.2	0.3
^214^Bi	502.4	4.8	10
^214^Pb	77.4	0.7	2
^226^Ra	0	0	1.4
^228^Ac	98.4	0.9	1.4
^234^Th	12.3	0.2	0
Sum	1807.3	17.2	61.1

**Table 3:** The equivalent H dose and the effective E dose obtained during a series of 15 peat mud paste applications to the skin by beta and gamma radiation during half-hour exposures.

Radionuclide	H	E (β)	E (γ)
	[nSv]		
^40^K	178.3	16.9	82.9
^137^Cs	78.8	0.75	2.5
^208^Tl	158.1	1.5	2.9
^210^Pb	0	0	0.3
^212^Bi	152.8	1.5	2.6
^212^Pb	30.9	0.3	0.5
^214^Bi	945.2	9	18.9
^214^Pb	141.4	1.3	3.7
^226^Ra	0	0	2.8
^228^Ac	189.6	1.8	2.7
^234^Th	23.9	0.2	0
Sum	1899	33.3	119.8

## References

[1] Chen Y., Stevenson F.J. (1986). Soil Organic Matter Interactions with Trace Elements.

[2] Schnitzer M. (1978). Humic Substances: Chemistry and reactions. Dev. Soil Sci..

[3] Senesi N. (1992). Metal-humic substance complexes in the environment. Molecular and Mechanistic Aspects by Multiple Spectroscopic Approach.

[4] Stevenson F.J. (1994). Humus Chemistry: Genesis, Composition, Reaction.

[5] Nero A.V., Schweher M.B., Nazaroff W.W., Revzan K.L. (1986). Distribution of Airborne Radon-222 Concentrations in USA Homes. Science.

[6] Spaccini R., Piccolo A., Haberhauer G., Stemmer M., Gerzabek M.H. (2001). Decomposition of maize straw in different European soils as revealed by DRIFT spectra of soil particle fractions. Geoderma.

[7] Kononova M.M. (1961). Soil organic matter. Its Nature, Its Role in Soil Formation and Soil Fertility.

[8] Pena-Mendez E.M., Havel J., Patocka J. (2005). Humic substances-compounds of still unknown structure: Application in agriculture, industry, environment and biomedicine. J. Appl. Biomed..

[9] Schulten H.R., Schnitzer M. (1997). Chemical model structures for soil organic matter and soils. Soil Sci..

[10] Thorn K.A., Goldenberg W.S., Youngerand S.J., Weber E.J., Gaffney J.S., Marley N.A., Clark S.B. (1996). Humic and Fulvic Acids: Isolation, Structure and Environmental Role.

[11] Borgman I.I. (1904). Radio-Activity in Russian mud baths. Lancet.

[12] Bostan B., Sen U., Gunes T., Sahin S.A., Sen C., Erdem M., Erkormaz U. (2010). Comparison of intra-articular hyaluronic acid injections and mud pack therapy in the treatment of knee osteoarthritis. Acta Orthop. Traumatol. Turc..

[13] Braghetta A., DiGiano F.A., Ball W.P. (1997). Nanofiltration of natural organic matter: pH and ionic strength effects. J. Environ. Eng..

[14] Brown G.K., MacCarthy L.J.A. (1999). Simultaneous determination of Ca, Cu, Ni, Zn and Cd binding strength with fulvic acid fraction Schubert’s method. Anal. Chim. Acta.

[15] Kulikova N., Stepanova E., Koroleva O., Perminova I.V., Hatfield K., Hertkorn N. (2005). Mitigating activity of humic substances: Direct influence on biota. Use of Humic Substances to Remediate Polluted Environments: From Theory to Practice. NATO Science Series (Series IV: Earth and Environmental Series).

[16] Joone G.K., van Rensburg C.E. (2004). An in vitro investigation of the anti-inflammatory properties of potassium humate. Inflammation.

[17] Junek R., Morrow R., Schoenherr J.I., Schubert R., Kallmeyer R., Phull S., Klocking R. (2009). Bimodal effect of humic acid on the LPS -induced TNF-alpha release from differentiated U937 cells. Phytomedicine.

[18] Van Rensburg C.E., Naude P. (2009). Potassium humate inhibits complement activation and the production of inflamaatory cytokines in vitro. Inflammation.

[19] Pazdzior S. (2013). Geological Documentation of the Therapeutic Peat Deposit.

[20] Currie L.A. (1968). Limits for qualitative detection and quantitative determination. Anal. Chem..

[21] Kocher D.C., Eckerman K.F. (1987). Electron dose-rate conversion factors for external exposure of the skin from uniformly deposited activity on the body surface. Health Phys..

[22] Yamaoka K., Mitsunobo F., Hanamoto K., Mori S., Tanizaki Y., Sugita K. (2004). Study on biologic effects of radon and thermal therapy on osteoarthritis. J. Pain.

[23] Franke A., Reiner L., Pratzel H.G., Franke T., Resch L.K. (2000). Long-term efficacy of radon spa therapy in rheumatoid arthritis-a randomized sham-controlled study and follow-up. Rheumatology.

[24] Ibrahim S.A., Li S.K. (2010). Chemical enhancer solubility in human stratum corneum lipids and enhancer mechanism of action on stratum corneum lipid domain. Int. J. Pharm..

[25] Kavasi N., Somlai J.G., Kovacs T., Schabo T., Varhegyi A., Hakl J. (2003). Occupational and patient doses in the therapeutic cave, Tapolca (Hungary). Radiat. Prot. Dosim..

[26] Somlai J., Torma A., Dobrovari P., Kavasi N., Nagy K., Kovacs T. (2007). Contribution of Rn-222, Ra-226, U-234 and U-238 radionuclides to the occupational and patient exposure in Heviz-spas in Hungary. J. Radioanal. Nucl. Chem..

[27] Tempfer H., Hofmann W., Schober A., Lettner H., Dinu A.L. (2010). Deposition of radon progeny on skin surfaces an resulting radiation doses in radon therapy. Radiat. Environ. Biophys..

[28] Walencik-Lata A., Kozlowska B., Dorda J., Przylibski T.A. (2016). The detailed analysis of natural radionuclides dissolved in spa waters of the Klodzko Valley, Sudety Mountains, Poland. Sci. Total Environ..

[29] Karpińska M., Mnich K., Kapała J., Bielawska A., Kulesza G., Mnich S. (2016). Radioactivity of peat mud used in therapy. J. Environ. Radioact..

[30] International Atomic Energy Agency (1996). International Basic Safety Standards for Protection against Ionizing Radiation and for the Safety of Radiation Sources.

[31] ICRP (1996). Age-Dependent Doses to Members of the Public from Intake of Radionuclides: Part 5 Compilation of Ingestion and Inhalation Dose Coefficients.

[32] ICRP (1991). Recommendation of the International Commission on Radiological Protection.

[33] ICRP (2007). The 2007 Recommendation of the International Commission on Radiological Protection.

[34] ICRP (2010). Conversion Coefficient for Radiological Protection Quantities for External Radiation Exposure, Annex, G. Special Considerations for Assessing the Local Skin-Equivalent Dose. Ann.

[35] Karpinska M., Mnich Z., Kapala J., Szpak A. (2009). The evaluation of indoor radon exposure in houses. Pol. J. Environ. Stud..

[36] The Regulation of the Minister of Health (2017). Reference Levels for Research and Treatments with the Use of Rentgen’s Radiation.

[37] Lehrer S., Rosenzweig K.E. (2015). Lung Cancer hormesis in high impact states where nuclear testing occurred. Clin. Lung Cancer..

[38] Rithidech K.N., Scott B.R. (2008). Evidence for radiation hormesis after in vitro exposure of human lymphocytes to low doses of ionizing radiation. Dose Response.

[39] Scott B.R. (2007). Low-dosses radiation-induced protective process and implications for risk assessment cancer prevention, and cancer therapy. Dose Response.

[40] Shi J., Huber M., Wang T., Dali W., Lin Z., Chun-Sheng Y. (2016). Progress in the studies on hormesis of low- dose pollutants. Environ. Dis..

[41] Vaiserman A.M. (2010). Radiation hormesis: Historical perspective and implications for low-dose cancer risk assessment. Dose Response.

[42] Yang G., Kong Q., Wang G., Jin H., Zhou L., Yu D., Niu C., Han W., Li W., Cui J. (2014). Low-dose ionizing radiation induced direct activation of natural killer cells and provides a novel approach for adoptive cellular immunotherapy. Cancer Biother. Radiopharm..

[43] Zhang F., Lin X., Yu L., Li W., Qian D., Cheng P., He L., Yang H., Zhang C. (2016). Low-dose radiation prevents type 1 diabetes-induced cardiomyopathy via activation of AKT mediated anti-apoptotic and anti-oxidant effects. J. Cell. Mol. Med..

[44] Bogdandi E.N., Balogh A., Felgyinszki N., Szatmari T., Persa E., Hildebrandt G., Safrany G., Lumniczky K. (2010). Effects low-dose radiation on the immune system of mice after total-body irradiation. Radiat. Res..

[45] Ina Y., Sakai K. (2005). Activation of immunological network by chronic low-dose irradiation in wild-type mouse strains: Analysis of immune cell populations and surface molecules. Int. J. Radiat. Biol..

[46] Liu X.D., Ma S.M., Liu S.Z. (2003). Effects of 0,075 Gy x-ray irradiation on the expression of IL-10 and IL-12 in mice. Phys. Med. Biol..

[47] Nowosielska E.M., Cheda A., Wrembel-Wargocka J., Janiak M.K. (2011). Anti-neoplastic and immunostimulatory effects of low dose X-ray fractions in mice. Int. J. Radiat. Biol..

[48] Pandey R., Shankar B.S., Sharma D., Sainis K.B. (2005). Low dose radiation induced immunomodulation: Effect on macrophages and CD8+ T cells. Int. J. Radiat. Biol..

[49] Sakai K., Nomura T., Ina Y. (2006). Enhancement of Bio-Protective Function by Low Dose/Dose-Rate Radiation. Dose Response.

[50] Shankar B., Sainis K.B. (2005). Cell cycle regulators modulating con A mitogenesis and apoptosis in low dose radiation-exposed mice. J. Environ. Pathol. Toxicol. Oncol..

